# The enteropathogenic *Escherichia coli* effector NleH inhibits apoptosis induced by *Clostridium difficile* toxin B

**DOI:** 10.1099/mic.0.037259-0

**Published:** 2010-06

**Authors:** Keith S. Robinson, Aurelie Mousnier, Cordula Hemrajani, Neil Fairweather, Cedric N. Berger, Gad Frankel

**Affiliations:** Centre for Molecular Microbiology and Infection, Division of Cell and Molecular Biology, Imperial College London, London SW7 2AZ, UK

## Abstract

*Clostridium difficile* is a leading cause of nosocomial infections, causing a spectrum of diseases ranging from diarrhoea to pseudomembranous colitis triggered by a range of virulence factors including *C. difficile* toxins A (TcdA) and B (TcdB). TcdA and TcdB are monoglucosyltransferases that irreversibly glycosylate small Rho GTPases, inhibiting their ability to interact with their effectors, guanine nucleotide exchange factors, and membrane partners, leading to disruption of downstream signalling pathways and cell death. In addition, TcdB targets the mitochondria, inducing the intrinsic apoptotic pathway resulting in TcdB-mediated apoptosis. Modulation of apoptosis is a common strategy used by infectious agents. Recently, we have shown that the enteropathogenic *Escherichia coli* (EPEC) type III secretion system effector NleH has a broad-range anti-apoptotic activity. In this study we examined the effects of NleH on cells challenged with TcdB. During infection with wild-type EPEC, NleH inhibited TcdB-induced apoptosis at both low and high toxin concentrations. Transfected *nleH1* alone was sufficient to block TcdB-induced cell rounding, nuclear condensation, mitochondrial swelling and lysis, and activation of caspase-3. These results show that NleH acts via a global anti-apoptotic pathway.

## INTRODUCTION

Enteropathogenic *Escherichia coli* (EPEC) and enterohaemorrhagic *E. coli* (EHEC) are closely related and important extracellular pathogens (Chen & Frankel, 2005; Nataro & Kaper, 1998) that while intimately adhering to gut enterocytes induce localized effacement of brush-border microvilli (Knutton *et al.*, 1987). Injection of bacterial effector proteins via a type III secretion system (T3SS) is an integral part of the EPEC and EHEC infection strategy (Frankel *et al.*, 1998; Garmendia *et al.*, 2005; Iguchi *et al.*, 2009; Tobe *et al.*, 2006) that reprogrammes cell signalling processes to facilitate colonization and infection (Garmendia *et al.*, 2005).

Among the conserved EPEC and EHEC effectors, Tir, EspG and Map subvert actin dynamics (Caron *et al.*, 2006), while EspF (McNamara *et al.*, 2001) disrupts the mitochondrial membrane potential and tight junctions. EspF also binds to and induces degradation of the anti-apoptotic protein AbcF2 (Nougayrede *et al.*, 2007). Importantly, EPEC-infected cells exhibit only early features of apoptosis, including expression of phosphatidylserine on the cell surface, and cleavage of cellular DNA and cytokeratin 18. However, EPEC-infected cells do not exhibit late apoptotic symptoms, including cell shrinkage, membrane blebbing or nuclear condensation and fragmentation (Crane *et al.*, 1999, 2001). As apoptosis relies on a fine balance between pro- and anti-apoptotic factors, we hypothesized the existence of effector(s) with anti-apoptotic activity, which neutralize the effects of EspF and promote cell survival. Indeed, we recently found that the EPEC T3SS effector NleH has a broad range of anti-apoptotic activities attributed to its interaction with the six-transmembrane endoplasmic reticulum (ER) protein Bax inhibitor 1 (Hemrajani *et al.*, 2010). We have shown that ectopic expression of NleH blocked apoptosis induced by the pro-apoptotic compounds staurosporine, a universal protein kinase inhibitor, and brefeldin A and tunicamycin, inducers of ER stress-related apoptosis (Hemrajani *et al.*, 2010). In particular, we have shown that expression of NleH prevented key apoptotic symptoms, including elevation of cytosolic Ca^2+^ concentration, membrane blebbing, nuclear condensation and activation of caspase-3.

*Clostridium difficile* is an anaerobic, spore-forming, Gram-positive pathogen and a leading cause of nosocomial infections (Bartlett *et al.*, 1978; George *et al.*, 1978; Rupnik *et al.*, 2009; Voth & Ballard, 2005). *C. difficile* is a commensal bacterium, frequently carried asymptomatically. However, antibiotic treatment of high-risk patients can alter the delicate balance within the gut microbiota, leading to sporulation and multiplication of this bacterium. *C. difficile* infection can cause a variety of symptoms ranging from diarrhoea to pseudomembranous colitis, which is associated with a high mortality rate (Kyne *et al.*, 2002).

The major virulence factors of *C. difficile* are the clostridial toxins A (TcdA) and B (TcdB) (Lyras *et al.*, 2009; Mani *et al.*, 2002), which are encoded by the *tcdA* and *tcdB* genes, respectively, located on a 19.6 kb pathogenicity locus termed PaLoc (Mani *et al.*, 2002; Matamouros *et al.*, 2007).

TcdA and TcdB are monoglucosyltransferases that irreversibly glycosylate small Rho GTPases, inhibiting their ability to interact with their effectors (Herrmann *et al.*, 1998; Sehr *et al.*, 1998), guanine nucleotide exchange factors (Herrmann *et al.*, 1998; Sehr *et al.*, 1998) and membrane partner (Genth *et al.*, 1999), leading to disruption of downstream signalling pathways and cell death (Just & Gerhard, 2004; Voth & Ballard, 2005). Targeting of TcdA and TcdB to Rho(A/B/C), RhoG, TC10, Rac1 and Cdc42 results in actin condensation, leading to dramatic rearrangement of the cytoskeleton, upregulation of pro-apoptotic factors (e.g. RhoB) and host cell rounding (Genth *et al.*, 2008; Voth & Ballard, 2005). Rho has a major role in stress fibre formation, motility and focal adhesions, whilst Rac1 and Cdc42 are involved in formation of filopodia and lamellipodia. It has been suggested that TcdB-treated cells require activation of the pro-apoptotic factor RhoB, via TcdB inactivation of RhoA, in order to induce the associated *C. difficile* cytotoxicity (Genth *et al.*, 2008). The cytopathic effects of TcdA and TcdB in tissue remain partially ambiguous, and the emergence of clinical isolates that are *tcdB^+^ tcdA^−^* but which result in clinical symptoms indistinguishable from *tcdA^+^ tcdB^+^* strains suggest that both toxins are not absolutely required for a productive infection (Kato *et al.*, 1998; Kuijper *et al.*, 2001; Limaye *et al.*, 2000). Recent work has indicated that *C. difficile*-induced apoptosis is due to TcdB inducing mitochondrial hyperpermeability via disruption of the mitochondrial membrane polarity, resulting in mitochondrial swelling, release of pro-apoptotic proteins and eventual lysis, all promoting progression of the host cell to apoptosis (Matarrese *et al.*, 2007). Apoptosis is executed via a number of different pathways, broadly differentiated into either extrinsic or intrinsic, with the pathways being either caspase dependent or independent. Caspases are cysteine proteases which are activated by cleavage in a hierarchical fashion and exist in the cytosol in zymogenic form. Once activated, caspases are important in cleaving key intracellular substrates, resulting in apoptosis (Budihardjo *et al.*, 1999). Bacterial virulence factors often target the mitochondrial membrane to modulate host cell death by permitting the release of pro-apoptotic proteins, e.g. cytochrome *c*. The aim of this study was to examine if NleH can inhibit TcdB-induced apoptosis.

## METHODS

### Bacterial strains and growth conditions.

Bacterial strains and plasmids used in this study are described in Table 1. Bacteria were grown at 37 °C in Luria–Bertani (LB) broth or agar supplemented with ampicillin (100 μg ml^−1^); chloramphenicol (25 μg ml^−1^) and kanamycin (50 μg ml^−1^) as appropriate.

EPEC bacteria were cultured in LB broth at 37 °C for 18 h with the required antibiotic. Overnight cultures were diluted at a ratio of 1 : 100 into colourless Dulbecco's Modified Eagle's Medium (DMEM), containing 1000 mg glucose l^−1^, to prime expression of the T3SS and effector proteins (Hemrajani *et al.*, 2010), and incubated for a further 3 h at 37 °C until the OD_600_ reached 0.3–0.35; strains containing pSA10-*nleH1* were induced for NleH expression with 1 mM IPTG for the final 30 min.

### *C. difficile* toxin B preparation.

Toxin B from *C. difficile* VPI10463 was prepared according to previously described methods Reineke *et al.* (2007) and was a gift from Dr C. von Eichel-Streiber. The toxin was examined by SDS-PAGE and deemed to be ≥95 % pure.

### Tissue culture and transfection.

HeLa cells were grown in DMEM containing 1000 mg glucose l^−1^ and supplemented with 10 % (v/v) fetal calf serum, non-essential amino acids and GlutaMAX in a humidified atmosphere at 5 % (v/v) CO_2_ at 37 °C. Cells were transfected with either pICC443 (pHM6-*nleH1*) or pEGFP-N1 (Clontech) (control plasmid) using lipofectamine 2000 (Invitrogen) in accordance with the manufacturer's protocol and incubated in a humidified atmosphere for 24 h before adding 4 ng TcdB ml^−1^; cells were then incubated for an additional 18 h. The transfection efficiency for pICC443 was ∼30–40 % whilst control plasmids were transfected at a higher efficiency of ∼70 % – this was controlled for during counting, with 100 transfected cells counted in a field of view.

### Cell viability assay.

HeLa cells were grown in 24-well plates with and without coverslips, washed and the growth medium replaced by colourless DMEM. Primed bacteria were normalized by optical density (OD_600_ 0.35) and used to infect for 1 h. Cells were washed five times with pre-warmed HBSS, treated with DMEM containing 200 μg gentamicin ml^−1^ (Invitrogen) to kill the bacteria, and 4 ng ml^−1^ or 4 μg ml^−1^ of TcdB was added for 18 or 4 h respectively. Cells were then washed three times in PBS (Sigma). Viability counting involved trypsinizing (300 μl) the cells, followed by neutralization with 700 μl DMEM, then viewing living cells using 0.05 % trypan blue in PBS mixed with cells at a ratio of 1 : 1 and counting in a Neubauer haemocytometer. All conditions were tested a minimum of three times and all counts were compared to the uninfected cells then plotted as a percentage of viable cells.

### Immunofluorescence and nuclear condensation.

Mitochondrial viability was tested using MitoTracker (Invitrogen) according to the manufacturer's guidelines before fixation. Cells were fixed in 3 % paraformaldehyde for 15 min at room temperature, washed, quenched for 30 min with 10 mM NH_4_Cl, permeabilized with 0.2 % Triton-X-100, washed again and incubated with 1 % BSA for 1 h. Caspase-3 activation was examined by staining overnight at 4 °C with monoclonal rabbit anti-cleaved caspase-3 (Cell Signalling Technology) and cells transfected with pICC449 (pHM6-*nleH1*, expressing an N-terminal HA tag) were visualized with mouse anti-HA (Covance) diluted 1 : 500 in 1 % BSA. Donkey anti-rabbit IgG conjugated to RRX and donkey anti-mouse conjugated to Cy2 (Jackson Laboratories) were diluted 1 : 200 in 1 % BSA for 45 min. Nuclear condensation was visualized by labelling DNA with Hoechst 33342 (Invitrogen) diluted 1 : 500 in 1 % BSA; bacterial presence was observed using rabbit anti-O127 diluted 1 : 500 and visualized with donkey anti-rabbit RRX (Jackson Laboratories) diluted 1 : 200 in 1 % BSA. Following antibody incubation, and a further three additional washes with PBS, the coverslips were mounted using ProLong Gold antifade reagent (Invitrogen) and visualized with a Zeiss Axioimager immunofluorescence microscope then analysed by Axiovision Rel 4.5 software.

### Statistics.

All statistical tests were done using GraphPad InStat Version 3.06 software on data from experiments done in triplicate and repeated a minimum of three times. Counts in experiments using transfected or infected cells were carried out as described above, with 100 cells counted in each repeat. The one-way ANOVA test using Bonferroni correction was used to determine significance.

## RESULTS

### NleH promotes cell survival by inhibiting TcdB-mediated cell death

**Fig. 1.**
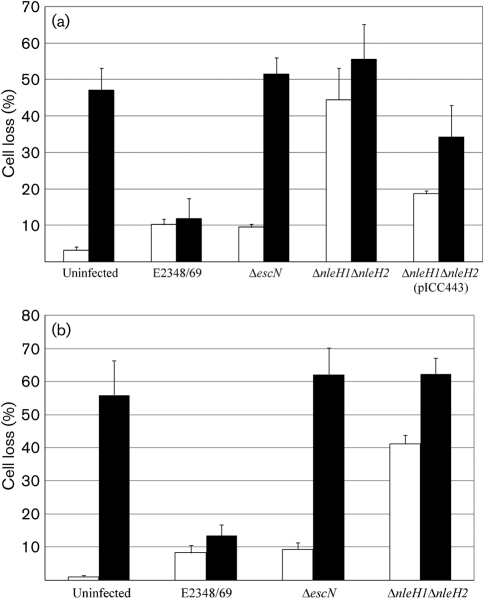
NleH inhibits *C. difficile* toxin B-mediated cell death. Cell viability counts were determined during infection of HeLa cells with wild-type EPEC, EPEC Δ*nleH1*Δ*nleH2* and the complemented strain Δ*nleH1*Δ*nleH2*(pICC443). Uninfected and EPEC Δ*escN*-infected cells were used as controls. Cells were left unchallenged (white bars) or challenged (black bars) with 4 ng TcdB ml^−1^ for 18 h (a) or 4 μg TcdB ml^−1^ for 4 h (b). In the absence of toxin, cell viability was significantly reduced following infection with EPEC Δ*nleH1*Δ*nleH2* compared with uninfected cells or cells infected with wild-type EPEC.

In order to examine if NleH has a cytoprotective activity in cells exposed to TcdB, HeLa cells were infected for 1 h with wild-type EPEC, EPEC Δ*escN* (T3SS-deficient mutant), EPEC Δ*nleH1*/Δ*nleH2* and EPEC Δ*nleH1*/Δ*nleH2* complemented with pICC443 (encoding NleH1) (Table 1). Following protein translocation and washes the attached bacteria were killed by the addition of gentamicin (200 μg ml^−1^). HeLa cells were challenged with 4 ng TcdB ml^−1^ for 18 h and the number of viable cells quantified (Fig. 1a). In the absence of toxin, infection with wild-type EPEC and the Δ*escN* mutant did not significantly alter cell survival. However, more than 40 % of cells did not survive EPEC Δ*nleH1*/Δ*nleH2* infection; *in trans* expression of NleH1 (pICC443) in the double mutant partially restored cell survival, as cell loss was reduced to <20 % (*P*<0.05) (Fig. 1a). Exposure to 4 ng TcdB ml^−1^ for 18 h resulted in a significant (>50 %) decrease in the number of living cells in uninfected control cultures and monolayers infected with EPEC Δ*escN* and EPEC Δ*nleH1*/Δ*nleH2* (*P*<0.05) (Fig. 1a). In contrast, infection with wild-type EPEC protected the monolayers, resulting in similar survival to that of wild-type infected cells without TcdB challenge. Complementation of the EPEC Δ*nleH1*/Δ*nleH2* mutant with pICC443 (encoding NleH1) resulted in partial cytoprotection (Fig. 1a). Importantly, the same pattern was observed when challenging HeLa cells infected with the same EPEC strains with 4 μg TcdB ml^−1^ for 4 h following the initial 1 h infection (*P*<0.001) (Fig. 1b). These results show that delivery of NleH during EPEC infection can protect cells from TcdB-induced cell death.

### NleH inhibits nuclear condensation

**Fig. 2.**
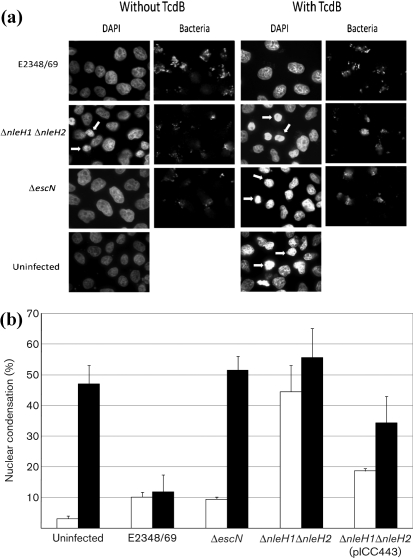
NleH prevents nuclear condensation induced by TcdB. HeLa cells were infected with wild-type EPEC, EPEC Δ*nleH1*Δ*nleH2* and complemented mutant and stained with Hoechst 33342 for evaluation of nuclear condensation or fragmentation; uninfected and EPEC Δ*escN*-infected cells were used as controls (a). The number of condensed nuclei (arrowed in a) was determined by counting under an epifluorescence microscope (b).

We determined if NleH can block TcdB-induced nuclear condensation. HeLa cells were infected with wild-type EPEC, EPEC Δ*escN*, EPEC Δ*nleH1*/Δ*nleH2* and EPEC Δ*nleH1*/Δ*nleH2* complemented with pICC443 (encoding NleH1). Hoechst staining was used to visualize nuclear condensation while bacterial adhesion was detected using anti-O127 antiserum. Examples of nuclei considered to be condensed are indicated by arrows in Fig. 2(a). Quantification of condensed nuclei by fluorescence microscopy showed that only 3 % of uninfected HeLa cells exhibited nuclear condensation, compared to 10 % and 9 % of cells infected with wild-type EPEC and EPEC Δ*escN*, respectively. In contrast, 44 % of the cells in monolayers infected with EPEC Δ*nleH1*Δ*nleH2* exhibited nuclear condensation, which is significantly higher (*P*<0.001) than the level seen in the other infected monolayers (Fig. 2b, white bars). Complementing the EPEC Δ*nleH1*Δ*nleH2* strain with pICC443 (encoding NleH1) reduced the frequency of cells with condensed nuclei to 19 % (Fig. 2b, white bars).

When challenged with 4 ng TcdB ml^−1^ TcdB for 18 h (Fig. 2b, black bars), uninfected HeLa cells (47 %) and cells infected with EPEC Δ*escN* (52 %) and EPEC Δ*nleH1*Δ*nleH2* (56 %) all showed a significant increase in nuclear condensation (*P*<0.05) compared to the same conditions in the absence of TcdB. However, HeLa cells infected with wild-type EPEC and challenged with 4 ng TcdB ml^−1^ showed no significant change in nuclear condensation, whilst cells infected with EPEC Δ*nleH1*Δ*nleH2* complemented with pICC443 (encoding NleH1) showed a significant difference (*P*<0.01) compared to uninfected, TcdB-untreated HeLa cells.

### NleH inhibits caspase-3 cleavage in the presence of TcdB

**Fig. 3.**
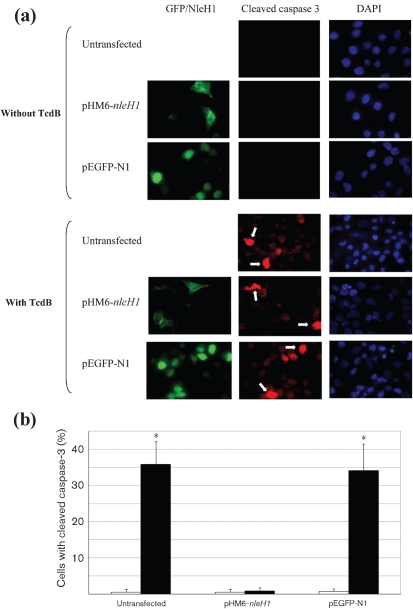
NleH1 prevents pro-caspase-3 cleavage induced by TcdB. Caspase-3 activation was determined in cells transfected with *nleH1* (pHM6-*nleH1*) or a *gfp* control (pEGFP-N1) and treated with 4 ng TcdB ml^−1^ for 18 h; unchallenged cells were used as a control. (a) Cells were stained with anti-HA (green) to label HA-tagged NleH1 and anti-cleaved caspase-3 (red) and visualized by immunofluorescence. (b) The level of caspase-3 cleavage in transfected cells was determined by counting under an epifluorescence microscope. Black bars, TcdB-treated cells; white bars, unchallenged controls. Expression of NleH1 significantly prevented the cleavage of pro-caspase-3 when cells were treated with TcdB compared to mock-transfected cells or cells transfected with pGFP.

TcdB-induced apoptosis is mediated by caspase-3 (Matarrese *et al.*, 2007). In order to investigate if NleH inhibits the cleavage of pro-caspase-3, HeLa cells were transfected with *nleH1* or with a *gfp* control and challenged with 4 ng TcdB ml^−1^ for 18 h. The number of cells with cleaved caspase-3 was assessed and quantified by immunofluorescence microscopy. Typical images are shown in Fig. 3(a); cells considered to have cleaved caspase-3 are indicated by arrows. Cells ectopically expressing *nleH1* did not show any difference in caspase-3 activation whether challenged with the toxin or not. However, both untransfected cells and the negative control (*gfp*-transfected cells) showed significantly higher caspase-3 activation (*P*<0.001) (Fig. 3b). This result suggests that NleH is capable of blocking TcdB-induced caspase-3 activation and therefore the progression of apoptosis.

### NleH prevents mitochondrial membrane disruption

**Fig. 4.**
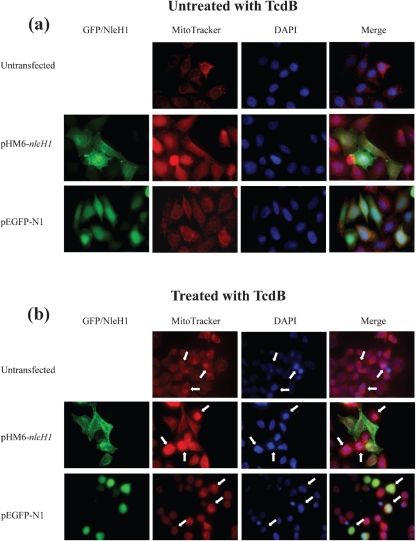
NleH1 prevents mitochondrial lysis in cells challenged with TcdB. Mitochondrial membrane potential was examined in cells transfected with *nleH1* (pHM6-*nleH1*) or a *gfp* control (pEGFP-N1) and treated or not with 4 ng TcdB ml^−1^ for 18 h. Cells were stained with anti-HA (green) to label N-terminally HA-tagged NleH1 and Mitotracker (Invitrogen) (red) and visualized by immunofluorescence (a). The loss of viable mitochondria in TcdB-treated cells is indicated by arrows in (b). Expression of NleH1 prevented the rupturing of mitochondria when cells were treated with TcdB compared to mock-transfected cells or transfected with the *gfp* control.

Recently it was reported that TcdB-induced apoptosis is due to hyperpermeable mitochondria (Matarrese *et al.*, 2007). We tested if NleH can enhance mitochondrial survival in cells challenged with TcdB. Mitochondrial viability was examined in HeLa cells transfected with *nleH1* or a *gfp* control and challenged with 4 ng TcdB ml^−1^ for 18 h. Mitochondrial viability was assessed using MitoTracker, a dye permeable to active mitochondria, and visualized by fluorescence microscopy; typical images are shown in Fig. 4. When cells were challenged with 4 ng TcdB ml^−1^ for 18 h, both untransfected cells and cells transfected with the *gfp* control exhibited loss of mitochondrial membrane potential, showing a condensed mitochondrial pattern (Fig. 4b, arrows). The mitochondria of cells transfected with *nleH1* were capable of taking up the MitoTracker dye, showing distinct mitochondria, whilst both untransfected cells and *gfp* controls showed uniform staining. This implies that NleH1 inhibits cytochrome *c* release in large quantities by inhibiting mitochondrial bursting, and/or that caspase-3 activation is inhibited and therefore the intrinsic pathway of apoptosis is not active in *nleH1*-transfected cells.

## DISCUSSION

It has been reported that the apoptotic effects of TcdB are potentially mediated in three different ways: firstly by inhibiting Rho GTPases, resulting in the inhibition of assembly of the actin cytoskeleton, which can lead to the loss of anchorage to the substratum and result in morphological changes related to apoptosis (Frankel *et al.*, 1998; Just *et al.*, 1995; Lyras *et al.*, 2009; Matarrese *et al.*, 2007; Reineke *et al.*, 2007); secondly, by disruption of downstream signalling events that Rho GTPases are involved in such as transcription factor activation/inactivation (Le *et al.*, 2005; Matarrese *et al.*, 2007); and thirdly, by disruption of the mitochondrial membrane potential, thereby inducing the activation of caspase-9 and ultimately caspases -3, -6 and -7 (Lyras *et al.*, 2009; Matarrese *et al.*, 2007).

The inhibition of Rho GTPases has a dual effect on actin assembly and downstream signalling events. Although the inactivation of Rho GTPases has not been fully investigated in this study it is evident that there is stabilization of the actin cytoskeleton at the plasma membrane. HeLa cells that were not transfected with *nleH1* largely showed signs of cell rounding following 18 h of exposure to TcdB (4 ng ml^−1^), a hallmark of TcdB challenge. However, cells expressing NleH1 maintained cell membrane integrity and did not show signs of rounding, suggesting that NleH1 plays a role in regulating critical anti-apoptotic pathways acting to curb the apoptotic actions of TcdB on Rho GTPase signalling and actin dynamics.

The third effect of TcdB is mitochondrial membrane hyperpolarization (MMHP), which was shown to be essential for TcdB-induced apoptosis (Matarrese *et al.*, 2007). TcdB was indicated to induce MMHP by blocking mitochondrial ATP-dependent potassium (_m_K_ATP_) channels, as activation of _m_K_ATP_ channels stopped TcdB-induced apoptosis (Matarrese *et al.*, 2007). An obvious phenotype of blocking _m_K_ATP_ channels is the rupturing of the mitochondrial membrane as a result of swelling (Desagher & Martinou, 2000). The induction of MMHP has been indicated as an early event in apoptosis (Gross *et al.*, 2000; Kroemer & Reed, 2000; Matarrese *et al.*, 2007) or results in the sensitization of cells to mitochondrial-related apoptosis (Kroemer & Reed, 2000; Matarrese *et al.*, 2003, 2007). This was not the case in *nleH1*-transfected cells, whose mitochondria were clearly not disrupted, suggesting that mitochondrial membrane polarity must still be intact; in addition they showed no signs of swelling. Supporting this, downstream of mitochondrial pro-apoptotic protein release, due to changes in mitochondrial membrane polarity or swelling and rupture, is the cleavage of caspase-3, which was not observed in *nleH1*-transfected cells. Therefore NleH must act prior to mitochondrial activation in the intrinsic apoptotic pathway, suggesting that the upstream regulation of either pro- or anti-apoptotic proteins is paramount.

It is also known that TcdB activation following endosomal lysis and release into the cytosol causes an increase in HeLa cell cytosolic Ca^2+^ levels, thought to be derived not from the ER Ca^2+^ store but from the extracellular environment (Matarrese *et al.*, 2007). It has been reported that the actions of TcdB are Ca^2+^ dependent, as the lack of Ca^2+^ in TcdB-intoxicated cells does not induce apoptosis (Matarrese *et al.*, 2007).

Recently we showed that NleH inhibits Ca^2+^ release from the ER due to interaction with Bax inhibitor 1 (BI-1) a known anti-apoptotic protein (Hemrajani *et al.*, 2010). Although Ca^2+^ was shown to come from the extracellular milieu for cells challenged solely with TcdB (Matarrese *et al.*, 2007), the regulation of Ca^2+^ uptake and cytoplasmic Ca^2+^ concentration is influenced by NleH, as infection with EPEC *nleH* mutants led to raised cytosolic Ca^2+^ levels (Hemrajani *et al.*, 2010). The inhibition by NleH of increasing a cell's cytoplasmic Ca^2+^ concentration could explain why TcdB does not induce MMHP and therefore apoptosis. However, the mechanism by which BI-1 inhibits apoptosis is not fully known, although it is known that it interacts with anti-apoptotic proteins Bcl-2 and Bcl-XL and blocks Bax-mediated apoptosis and that its overexpression prevents chemically induced apoptosis by staurosporine, tunicamycin, brefeldin A and thapsigargin (Chae *et al.*, 2004; Xu & Reed, 1998). The ability of BI-1 to inhibit the induction of many forms of apoptosis could be at the heart of how NleH1 prevents cell death from TcdB.

It is known that NleH effectors possess multiple domains, one of which is a kinase domain (Gao *et al.*, 2009; Hemrajani *et al.*, 2010). We have shown that the loss of this domain does not affect the anti-apoptotic function of NleH effectors either in the effector repertoire during EPEC infection or during ectopic NleH expression when cells are challenged with the pro-apoptotic compounds staurosporine, tunicamycin and brefeldin A (Hemrajani *et al.*, 2010). In addition, Gao *et al.* (2009) showed the relevance of the first 100 amino acids of NleH effectors in binding to the ribosomal protein S3 (Rps3), a subunit of the nuclear factor kappa-light-chain enhancer of activated B cells (NF*κ*B), regulating NF*κ*B transcriptional activity. They showed that NleH1 reduced the nuclear abundance of Rps3, inhibiting NF*κ*B activity.

NF*κ*B plays a key role in regulating the immune response to infection and anti-apoptotic genes. It was shown that the loss of *nleH1* from EHEC O157 : H7 produced a hypervirulent form in gnotobiotic piglets, resulting in premature death (Gao *et al.*, 2009). This finding would suggest that NleH1 itself is important for pathogenesis and that it has relevance both to the clinical characteristic of EHEC and EPEC infection and to proliferation in the host. Furthermore, the anti-apoptotic effect of NleH1 is likely to occur independently of the NF*κ*B pathway, as its inhibition could reduce the transcription of anti-apoptotic host genes.

In this study we have confirmed the ability of TcdB to trigger apoptosis. The induction of apoptosis by TcdB is likely to be due to a number of different actions including inhibition of Rho GTPases inducing cell rounding as well as mitochondrial hyperpermeability stimulating the intrinsic apoptotic pathway. We have already shown that NleH can block apoptosis induced by staurosporine, tunicamycin and brefeldin A; its ability also to block TcdB-induced apoptosis suggests that NleH targets a basic mechanism common to these unrelated apoptosis pathways by acting on a global anti-apoptotic pathway, independent of the NF*κ*B pathway, that is yet to be determined.

## Figures and Tables

**Table 1.** Strains and plasmids

**Strain or plasmid**	**Characteristics**	**Source/reference**
**Strains**		
E2348/69	Wild-type EPEC O127:H6	Levine *et al.* (1978)
ICC217	E2348/69 Δ*escN, escN* : : Kan (Kan^r^)	Garmendia *et al.* (2004)
ICC303	E2348/69 Δ*nleH1*Δ*nleH2*, *nleH1* : : Kan, *nleH2* : : Cm (Kan^r^, Cm^r^)	Hemrajani *et al.* (2010)
**Plasmids**		
pSA10	pKK177-3 expression vector containing *lacI* gene	Schlosser-Silverman *et al.* (2000)
pICC443	pSA10-*nleH1*, derivative of pSA10, expressing NleH1	Hemrajani *et al.* (2010)
pHM6	Mammalian expression vector, N-term HA tag and C-term His-tag	Roche
pICC449	pHM6-*nleH1*, derivative of pHM6, expressing HA-NleH1, stop codon before His-tag	Hemrajani *et al.* (2010)
pEGFP-N1	Mammalian GFP expression vector	Clontech

## Abbreviations

- EHEC, enterohaemorrhagic *E. coli*
- EPEC, enteropathogenic *Escherichia coli*
- ER, endoplasmic reticulum
- MMHP, mitochondrial membrane hyperpolarization
- T3SS, type III secretion system
